# Incidence of serious morbidity in HIV-infected adults on antiretroviral therapy in a West African care centre, 2003-2008

**DOI:** 10.1186/1471-2334-13-607

**Published:** 2013-12-27

**Authors:** Yao Abo, Albert Minga, Hervé Menan, Christine Danel, Timothée Ouassa, Lambert Dohoun, Germain Bomisso, Anthony Tanoh, Eugène Messou, Serge Eholié, Charlotte Lewden, Xavier Anglaret

**Affiliations:** 1 Programme PAC-CI, Centre Hospitalier Universitaire (CHU) de Treichville, 18 BP 1954, Abidjan, Côte d’Ivoire; 2 Centre de Diagnostic et de Recherche sur le SIDA et les Maladies Opportunistes (CeDReS), Centre Hospitalier Universitaire (CHU) de Treichville, Abidjan, Côte d’Ivoire; 3 INSERM, Centre INSERM U897-Epidémiologie-Biostatistique, Bordeaux, France; 4 Univ. Bordeaux, ISPED, Bordeaux, France; 5 Fondation Ariel Glaser pour la lutte contre le SIDA Pédiatrique, Abidjan, Côte d’Ivoire; 6 Centre de Prise en charge de Recherche et de Formation (CePReF), Hôpital Yopougon Attié, Abidjan, Côte d’Ivoire; 7 Service de Maladies Infectieuses et Tropicales, Centre hospitalier Universitaire (CHU) de Treichville, Abidjan, Côte d’Ivoire

**Keywords:** Antiretroviral therapy, Serious morbidity, Routine care, Côte d’Ivoire

## Abstract

**Background:**

In resource-limited settings, scaling-up antiretroviral treatment (ART) has required the involvement of decentralized health facilities with limited equipment. We estimated the incidence of serious morbidity among HIV-infected adults receiving ART in one of these HIV routine care center in sub-Saharan Africa.

**Methods:**

We conducted a prospective study at the Centre Medical de Suivi des Donneurs de Sang (CMSDS), which is affiliated with the National Centre for Blood Transfusion in Abidjan, Côte d’Ivoire. Adult patients infected with HIV-1 or HIV-1/HIV-2 who initiated ART between January 2003 and December 2008 were eligible for the study. Standardized clinical data were collected at each visit. Serious morbidity was defined as a new episode of malaria, WHO stage 3–4 event, ANRS grade 3–4 adverse event, or any event leading to death or to hospitalization.

**Results:**

1008 adults, 67% women, with a median age of 35 years, and a median pre-ART CD4 count of 186/mm^3^ started ART and were followed for a median of 17.3 months. The overall incidences of loss to follow-up, death, and attrition were 6.2/100 person-years (PY) [95% CI 5.1-7.2], 2.3/100 PY [95% CI 1.6-2.9], and 8.1/100 PY [95% CI 7.0-9.4], respectively. The incidence of first serious event was 11.5/100 PY overall, 15.9/100 PY within the first year and 8.3/100 PY thereafter. The most frequently documented specific diagnoses were malaria, tuberculosis, bacterial septicemia and bacterial pneumonia.

**Conclusion:**

Among HIV-infected adults followed in routine conditions in a West African primary care clinic, we recorded a high incidence of serious morbidity during the first year on ART. Providing care centers with diagnostic tools and standardizing data collection are necessary steps to improve the quality of care in primary care facilities in sub-Saharan Africa.

## Background

Since 2004, through the combined effort of national institutions and international organizations including the World Health Organization (WHO), the Global Fund Against AIDS, Tuberculosis and Malaria, and the U.S President’s Emergency Plan for AIDS Relief (PEPfAR), antiretroviral therapy (ART) has become increasingly more available in resource-limited setting [1]. The wide distribution of ART has recently led to a major reduction in morbidity and mortality associated with Human Immunodeficiency Virus (HIV) infection in sub-Saharan Africa [2,3]. However, severe morbidity and mortality remain frequent, especially during the first year on ART [4-7]. Severe events mostly occur in a context of (i) late diagnosis of HIV infection and late initiation of ART [2]; (ii) high incidence of both opportunistic infections and communicable diseases such as tuberculosis (TB), bacterial diseases, and malaria [8,9] and (iii) scarce diagnostic tools and limited resources for patients [10,11].

Data regarding morbidity in HIV-infected adults on ART in Africa come mainly from research studies in selected populations that have free access to care. However, because scaling-up ART requires the involvement of decentralized health facilities for HIV case management, many facilities that provide HIV care are only primary care clinics and are often under equipped and lack resources for adequate diagnoses [12]. In these settings, patients followed under routine conditions have to pay for most tests and drugs. Thus, some patients for whom the cost of care is not affordable don’t access care. As a result, the frequency and spectrum of severe morbidity are partially unknown [13].

In this study, we estimated the incidence and spectrum of serious morbidity events among HIV-infected adults receiving ART in a HIV routine care center in Côte d’Ivoire, West Africa. Patients were not followed as part of a research study and had the same access to diagnostic tests as any other patients enrolled in the national HIV program.

## Methods

### Study population

This prospective study was conducted at the Centre Medical de Suivi des Donneurs de Sang (CMSDS), a urban care center affiliated with the National Centre for Blood Transfusion in Abidjan, Côte d’Ivoire. Patients infected with HIV-1 or HIV-1/HIV-2, 18 years or older, who initiated ART between January 1^st^, 2003 and December 31^st^, 2008, were eligible for this study. During the study period, ART was provided in this centre according to the Côte d’Ivoire national guidelines, adapted from the 2006 WHO guidelines for ART: CD4 count < 200 cells/mm^3^, WHO clinical stage 4 irrespective of CD4 count, or WHO clinical stage 3 with a CD4 count < 350 cells/mm^3^[14]; and cotrimoxazole prophylaxis was given to all patients with a CD4 count < 500 cells/mm^3^. Isoniazide Prophylaxis for Tuberculosis was not given, since it was not allowed by the Côte d’Ivoire National TB program.

After initiation of ART, patients were asked to attend scheduled visits at one week, two weeks (only in nevirapine-containing regimen), every month during the first three months, and every two months thereafter. Standardized clinical data were collected at each follow-up visit and biological data every 6 months. In Côte d’Ivoire, routine pre-art tests include cell blood count, CD4 cell count, glycemia plasma creatinine and plasma transaminases; and routine follow-up tests for patients on ART include cell blood count and CD4 cell count every six months.

Unscheduled visits were possible at any time during working hours in case of illnesses requiring consultation. The centrer was opened Monday to Friday from 7:30 to 5:00 p.m, and had a day-care hospital for patients with serious morbidity whose condition allowed them to be treated overday and spend the night at home. This included conditions requiring repeated overday care over a long period. Patients whose condition required overnight care were referred to the nearby Treichville university hospital. All data collected within the study center were prospectively entered into the center computer database. For patients who were referred for hospitalization, data were retrospectively collected using a standardized form. ART, CD4 counts, and cotrimoxazole prophylaxis were free. The cost of other tests and drugs was borne by patients.

Patients who did not show up for scheduled visits were contacted by telephone or visited at home, provided they had given consent to the care center team doing so at their first visit. After the study closing date (December 31^st^, 2008), this tracing procedure was extended up to August 31^st^ 2009. All patients whose last contact with the study center was prior to 31 Dec 2008, and who were not found to be deceased, alive, or transferred out up to August 31^st^ 2009, were defined as lost to follow-up.

The CMSDS is a participating center in the International epidemiological Database to Evaluate AIDS (IeDEA) in West Africa.

### Case definition of serious morbidity

The following events were considered serious morbidity events: (i) any WHO stage 3 or 4 classifying episodes [15]; (ii) any grade 3 or 4 classifying adverse events, except neutropenia for which only grade 4 events were considered; adverse events were graded according to the French National Agency for Research on AIDS and viral hepatitis (ANRS) severity scale for scoring [16]; (iii) any event leading to a hospital stay of one day or more; (iv) all documented episodes of malaria; (v) any event leading to death.

The diagnostic criteria were standardized and identical to those used for the Primo-CI cohort, a cohort of HIV-infected seroconverters followed up under the supervision of this same team. Methods are described elsewhere [8]. In summary, malaria was defined as a consistent clinical picture with a positive Quantitative Buffy Coat (QBC) test and/or a positive blood film for malaria by using May-Grünwald-Giemsa staining. The following bacterial diseases were regarded as serious and therefore classified as WHO clinical stage 3: bacteraemia, pneumonia, pleurisy, enteritis with stool cultures positive for a clinically significant bacteria, salpyngitis, pyelonephritis, prostatitis, orchi-epididymitis, meningitis, endocarditis, pyomyositis, pericarditis, or deep abscess. Active TB was defined in 3 different ways. “Definitive” TB was defined as a consistent clinical picture and positive culture for *Mycobacterium tuberculosis* identified in sputum samples or other extra-pulmonary fluids or tissue cultures; “presumptive” TB as a consistent clinical picture, with evidence of acid-fast resistant bacilli (or typical appearance in immunofluorescence) in sputum sample or other extra-pulmonary liquids and tissues; and “*possible*” TB as a consistent clinical picture for >30 days, with no criteria met for definitive or probable TB, no improvement after non-specific broad-spectrum antibiotic treatment, and for which medical investigators decide to initiate treatment. Bacterial pneumonia was defined as consistent clinical picture, with chest radiographic evidence of alveolar pulmonary disease, and: (i) isolation of a clinically significant bacterial pathogen from blood culture (definitive bacterial pneumonia) or (ii) successful response to antibiotherapy with no activity against *Pneumocystis carinii* (presumptive bacterial pneumonia).

The only point-of-care test available at our center was the QBC test for malaria. For all other tests, the specimens had to be sent to the Trecihville hospital laboratory.

### Statistical analysis

First, for all serious morbidity events, we reported the overall number of episodes that occurred during follow-up, including first episode and recurrent episodes. Then, we estimated the incidence rate of the first episode. We also estimated the incidence of death, loss to follow-up, and attrition (death or loss to follow-up). We compared the incidence of first episode of severe morbidity, death, loss to follow-up, and attrition, between pre-ART CD4 groups (<100, 100–200, >200/mm^3^) and periods of ART (0–12, >12 months) using univariable Poisson models. Finally, we estimated the probability of death, loss to follow-up, attrition, and first severe morbidity episode, using the Kaplan-Meier method. Data were shown with their 95% confidence interval (CI). For each analysis of a given event, the “at-risk period” began at the date of ART initiation and ended on the day when the first event occurred or on December 31^st^, 2008.

We used SAS version 9.1 (SAS Institute, Cary North Carolina, USA) to perform the analysis.

### Ethical approval

The national ethics committee of Côte d’Ivoire gave an ethical approval for the use of all routinely collected data for the care and management of HIV-positive patients followed in the IeDEA West Africa collaboration. Written informed consent was obtained from all participants at the Centre Medical de Suivi des Donneurs de Sang (CMSDS).

## Results

### Patients, follow-up, and number of events

Among 1182 adults who were followed on ART in our center between January 2003 and December 2008, 174 had started ART prior to January 1^st^ 2003, and 1008 started ART during the study period (2003: n = 100; 2004: n = 82; 2005: n = 130; 2006: n = 168; 2007: n = 201, 2008: n = 327). Of these patients, 67% were female, and median age was 35 years (interquartile range [IQR] 30–42). 96% of patients were infected with HIV-1 alone, 87% were at WHO clinical stage I or II at ART initiation and 70% had received cotrimoxazole prophylaxis at least once prior to ART initiation. The most frequently prescribed ART regimens were d4T, 3TC and nevirapine (48%) and AZT, 3TC and efavirenz (23%). At ART initiation, the median body mass index (BMI) was 21.3 kg/m^2^ (IQR 19.1-23.9) and the median CD4 count 186/mm^3^ (IQR 97–259). The cumulative follow-up from the time of ART initiation to the end of study date was 2076 person-years (PY), with a median of 17.3 months (IQR 5.7-38.2) (Table 1).

**Table 1:** Patients pre-ART and on-ART follow-up characteristics, CMSDS, Abidjan, Côte d’Ivoire, 2003-2008

**Characteristics**	**N = 1008**	
**At ART initiation**		
Sex, female, n (%)	673	(67)
Age (years), median (IQR)	35.0	(29.7-41.9)
CD4 count (cell/mm^3^), median (IQR)	186	(97–258)
WHO clinical stage 3 or 4, n (%)	136	(13)
BMI (kg/m^2^), median (IQR)	21.3	(19.1-23.9)
Patients on specific ART regimen, n (%)		
2NRTIs + nevirapine ^α^	562	(55.8)
2NRTIs + efavirenz ^β^	332	(32.9)
2NRTIs + ritonavir boosted PI*	78	(7.7)
Others**	36	(3.6)
Hemoglobin (g/dl), median (IQR)	10.8	(9.4-12.1)
Patients with prior use of cotrimoxazole^†^, n (%)	709	(70)
**Follow-up on ART**		
Cumulative (PY)	2076	
Median (IQR), in months	17.3	(5.7-38.0)
Death, n (%)	47	(4.6)
Patients lost to follow-up^‡^, n (%)	128	(12.7)

*IQR*, Interquartile Range; *WHO*, World Health Organization; *ART*, Antiretroviral Therapy; *BMI*, Body Mass Index; *d4T*, Stavudine, *3TC*, Lamivudine; *AZT*, zidovudine; *ddI*, Didanosine; *TDF*, Tenovofir diproxil fumarate; *FTC*, Emtricitabine; *ABC*, Abacavir.

α: d4T + 3TC (n = 484), AZT + 3TC (n = 78).

β: AZT + 3TC (n = 234), d4T + 3TC (n = 56), TDF + FTC (n = 38), d4T + ddI (n = 2), ddI + 3TC (n = 1), ABC + 3TC (n = 1).

*: AZT + 3TC (n = 25), TDF + FTC (n = 12), d4T + 3TC (n = 4), ddI + ABC (n = 4), ddI + 3TC (n = 1), ABC + 3TC (n = 1)] + lopinavir/ritonavir (n = 47); AZT + 3TC (n = 21), d4T + 3TC (n = 10)] + indinavir/ritonavir (n = 31).

**: 3NRTIs (n = 30) : TDF + FTC + AZT (n = 28), AZT + 3TC + ABC (n = 2); Non-boosted PIs (n = 6) : AZT + 3TC + indinavir (n = 3), AZT + 3TC + nelfinavir (n = 3).

†: Use of cotrimoxazole at least once prior to antiretroviral initiation.

‡: Lost to follow-up: patients whose last contact with the study center was prior to 31 Dec 2008, and who were not found to be deceased, alive, or transferred out up to August 31^st^ 2009.

Overall, 278 serious events were recorded in 192 patients. These were WHO stage 3 or 4 events (61 episodes, 54 patients), confirmed malaria (87 episodes, 63 patients), ANRS grade 3 or 4 events (49 episodes, 45 patients), and other events leading to hospitalization or death (81 episodes, 79 patients) (Table 2).

**Table 2:** Details of the 278 serious morbidity events recorded after ART initiation, CMSDS, Abidjan, Côte d’Ivoire, 2003-2008

	**Overall**		**WHO stage 3 or 4**		**ANRS grade 3 or 4**	
**Parasitic diseases, n (%)**	**90**	**(32.4)**				
Confirmed malaria	87	**–**	**–**	**–**	**–**	**–**
Parasitic enteritis	3	**–**	**–**	**–**	**–**	**–**
**Mycobacterial diseases, n (%)**	**33**	**(11.9)**				
Tuberculosis	33	**–**	33	**–**	**–**	**–**
**Bacterial diseases, n (%)**	**14**	**(5)**				
Septicemia	5	**–**	5	**–**	**–**	**–**
Pneumonia	5	**–**	5	**–**	**–**	**–**
Bacterial enteritis	2	**–**	**–**	**–**	**–**	**–**
Pyelonephritis	2	**–**	2	**–**	**–**	**–**
**Fungal diseases, n (%)**	**4**	**(1.4)**				
Esophageal candidiasis	1	**–**	1	**–**	**–**	**–**
Oral candidiasis	3	**–**	3	**–**	**–**	**–**
**Hematologic diseases, n (%)**	**39**	**(14)**				
Anemia	19	**–**	**–**	**–**	19	**–**
Neutropenia	18	**–**	**–**	**–**	18	**–**
Thrombopenia	2	**–**	**–**	**–**	2	**–**
**Others**^**†**^**, n (%)**	**50**	**(18)**				
Unexplained acute fever	14	**–**	**–**	**–**	**–**	**–**
Unexplained acute diarrhea	7	**–**	**–**	**–**	**–**	**–**
Unexplained prolonged fever	6	**–**	6	**–**	**–**	**–**
Hyperlactatemia	5	**–**	**–**	**–**	5	**–**
Unexplained pulmonary disease	3	**–**	**–**	**–**	**–**	**–**
Elevated liver enzymes	2	**–**	**–**	**–**	2	**–**
Toxiderma	2	**–**	**–**	**–**	2	**–**
Acute delusional psychosis	1	**–**	**–**	**–**	1	**–**
Herpes Zoster	1		**–**	**–**	**–**	**–**
Hypertension	1		**–**	**–**	**–**	**–**
Miscellaneous	8		**–**	**–**	**–**	**–**
**Death, n (%)**	**47**	**(16.9)**	**–**	**–**	**–**	**–**
**Total**	**278**	**(100)**	**61**	**(21.9)**	**49**	**(17.6)**

*WHO*, World Health Organization; *ANRS*, Agence Nationale de Recherche sur le SIDA et les Hépatites Virales.

Septicemia: consistent clinical picture and clinically significant pathogen isolated from blood culture

Pneumonia: Pulmonary symptoms, fever, chest radiographic evidence of alveolar pulmonary disease and : (i) successful response to antibiotherapy with no activity against *Pneumocystis carinii*; or (ii) clinically significant bacterial pathogen isolated from blood culture.

Parasitic enteritis: diarrhea and clinically significant parasitic pathogen on stool examination.

Bacterial enteritis: diarrhea and clinically significant bacterial pathogen isolated from stool culture.

†: non-classifying events with ≥ one day at hospital, or leading to death.

Unexplained acute fever: Fever > 37.5°C of unexplained origin for < 30 days.

Unexplained prolonged fever: Fever > 37.5 of unexplained origin for ≥ 30 days, intermittent or constant.

Unexplained pulmonary disease: pulmonary fibrosis, n = 1; mediastinal mass, n = 1.

Zoster: patient with zoster of the ear canal, fever and meningeal syndrome. Negative blood films for malaria, negative blood culture and negative cerebrospinal fluid culture.

### Incidences and probabilities of severe morbidity, death, and LTFU

**Figure 1:**
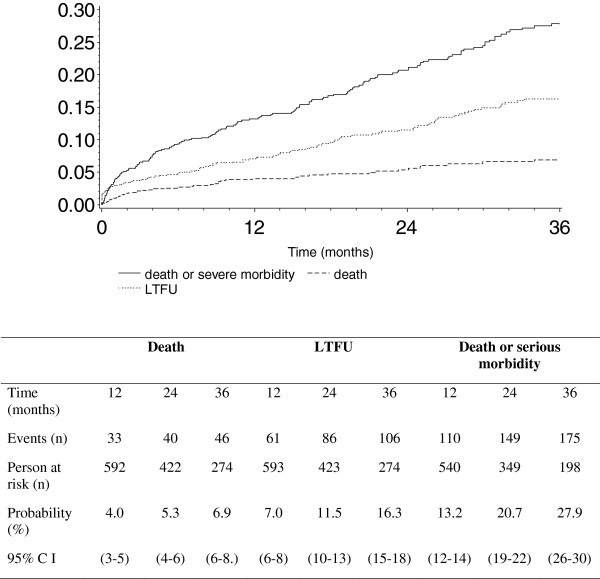
**Cumulative probability of death or serious morbidity following ART initiation; CMSDS, Abidjan, Côte d’Ivoire, 2003-2008.** CI = Confidence Interval. Lost to follow-up: patients whose last contact with the study center was prior to 31 Dec 2008, and who were not found to be deceased, alive, or transferred out up to August 31^st^ 2009.

The incidence of serious morbidity, death, loss to follow-up, and attrition was 11.5/100 PY (95% CI 9.9-13.1), 2.3/100 PY (95% CI: 1.6-2.9), 6.2/100 PY (95% CI 5.1-7.2), and 8.1/100 PY [95% CI 7.0-9.4], respectively. Serious morbidity was more frequent within the first year of treatment, and in patients with pre-ART CD4 counts below 100/mm^3^ (Table 3 and Table 4). Figure 1 shows the cumulative probabilities of death, serious illness and/or loss-to-follow-up over time.

**Table 3:** Incidence of first serious morbidity event, death, loss to follow-up and attrition following ART initiation, by time on ART; CMSDS, Abidjan, Côte d’Ivoire, 2003-2008

	**Duration of follow-up (months)**				P **
	**0 to 12**		**12 to 72**		
**Serious morbidity**					
Overall					10^-4^
Events, n (Time at risk, PY)	111	(698)	81	(972)	
Incidence/100 PY (95% CI)	15.9	(13.0-18.9)	8.3	(6.5-10.2)	
WHO stage III or IV events					4.10^-3^
Events, n (Time at risk, PY)	28	(734)	20	(1214)	
Incidence/100 PY (95% CI)	3.8	(2.4-5.2)	1.7	(0.9-2.4)	
ANRS grade 3 or 4					6.10^-3^
Events number (Time at risk, PY)	26	(740)	19	(1233)	
Incidence/100 PY (95% CI)	3.5	(2.2-4.9)	1.5	(0.8-2.2)	
Confirmed malaria					0.80
Events number (Time at risk, PY)	24	(737)	39	(1124)	
Incidence/100 PY (95% CI)	3.3	(2.0-4.6)	3.5	(2.4-4.6)	
Other serious events*					5.10^-3^
Events number (Time at risk, PY)	22	(741)	15	(1268)	
Incidence/100 PY (95% CI)	3.0	(1.7-4.2)	1.2	(0.6-1.8)	
**Death**					10^-4^
Events number (Time at risk, PY)	32	(751)	15	(1324)	
Incidence/100 PY (95% CI)	4.3	(2.8-5.7)	1.1	(0.6-1.7)	
**Lost to follow-up**					10^-2^
Events number (Time at risk, PY)	61	(778)	67	(1371)	
Incidence/100 PY (95% CI)	7.8	(5.9-9.8)	4.9	(3.7-6.1)	
**Attrition**					10^-4^
Events number (Time at risk, PY)	95	(769)	80	(1369)	
Incidence/100 PY (95% CI)	12.4	(9.9-14.8)	5.8	(4.6-7.1)	

*PY*, Person-Years; *CI*, Confidence Interval; *ART*, Antiretroviral Therapy; *WHO*, World Health Organization; *ANRS*, Agence Nationale de Recherche sur le SIDA et les Hépatites Virales.

*: non-classifying events with ≥ one day at hospital, or leading to death.

Lost to follow-up: patients whose last contact with the study center was prior to 31 Dec 2008, and who were not found to be deceased, alive, or transferred out up to August 31^st^ 2009.

**: univariable Poisson models.

**Table 4:** Incidence of first serious morbidity event, death, loss to follow-up and attrition following ART initiation, by pre-ART CD4 strata;, CMSDS, Abidjan, Côte d’Ivoire, 2003-2008

	**CD4 count at ART initiation (cell/mm**^**3**^**)**						P **
	**< 100**		**100 to 200**		**> 200**		
**Serious morbidity**							
Overall							10^-2^
Events, n (Time at risk/100 PY)	58	(348)	49	(474)	85	(850)	
Incidence/100 PY (95% CI)	16.7	(12.4-20.9)	10.4	(7.5-13.3)	10.0	(7.9-12.1)	
WHO stage III or IV events							0.40
Events number (Time at risk/100 PY)	13	(391)	15	(549)	21	(1009)	
Incidence/100 PY (95% CI)	3.1	(1.3-4.8)	2.7	(1.4-4.1)	2.1	(1.2-3.0)	
ANRS grade 3 or 4							0.60
Events number (Time at risk/100 PY)	11	(392)	10	(558)	24	(1022)	
Incidence/100 PY (95% CI)	2.8	(1.2-4.5)	1.8	(0.7-2.9)	2.4	(1.4-3.3)	
Confirmed malaria							2.10^-2^
Events number (Time at risk/100 PY)	6	(381)	15	(529)	42	(968)	
Incidence/100 PY (95% CI)	1.6	(0.3-2.8)	2.8	(1.4-4.3)	4.3	(3.0-5.7)	
Other events*							7.10^-2^
Events number (Time at risk/100 PY)	13	(387)	9	(562)	15	(1060)	
Incidence/100 PY (95% CI)	3.4	(1.5-5.2)	1.6	(0.6-2.7)	1.4	(0.7-2.1)	
**Death**							10^-4^
Events number (Time at risk/100 PY)	26	(399)	13	(575)	8	(1100)	
Incidence/100 PY (95% CI)	6.5	(4.0-9.0)	2.3	(1.0-3.5)	0.7	(0.2-1.2)	
**Lost to follow-up**							10^-4^
Events number (Time at risk/100 PY)	40	(415)	47	(596)	41	(1138)	
Incidence/100 PY (95% CI)	9.6	(6.7-12.6)	7.9	(5.6-10.1)	3.6	(2.5-4.7)	
**Attrition**							10^-4^
Events number (Time at risk/100 PY)	66	(412)	70	(592)	49	(1132)	
Incidence/100 PY (95% CI)	16.0	(12.1-19.9)	11.8	(9.1-14.6)	4.3	(3.1-5.5)	

*PY*, Person-Years; *CI*, Confidence Interval; *ART*, Antiretroviral Therapy; *WHO*, World Health Organization; *ANRS*, Agence Nationale de Recherche sur le SIDA et les Hépatites Virales.

*: non-classifying events with ≥ one day at hospital, or leading to death.

Lost to follow-up: patients whose last contact with the study center was prior to 31 Dec 2008, and who were not found to be deceased, alive, or transferred out up to August 31^st^ 2009.

**: univariable Poisson models.

The strains isolated in blood culture from 5 patients with bacterial septicemia were *non-typhi Salmonella* (n = 3), *Streptococcus pneumoniae* (n = 1), and *Acinetobacter baumanii* (n = 1). Of the 87 episodes of malaria, 66 were confirmed by the QBC test and 21 by blood film. In the latter, the mean parasitemia was 11,551 trophozites/μL (SD 9.979). The three parasitis enteritis were caused by *Giardia intestinalis* and the two bacterial enteritis by *non-typhi Salmonella. Escherichia coli* was isolated from urine culture in one of the two episodes of pyelonephritis. The other patient had consistent clinical symptoms with a leukocyturia above one million per milliliter but negative urine culture. Mean temperature in acute febrile episodes without final specific diagnosis was 39.1°C (SD 0.6). All patients with severe anemia were transfused. A woman with a body weight of 21 kilos who was prescribed 600 mg per day of efavirenz suffered from acute delusional psychosis. Symptoms stopped after a dose reduction to 400 mg per day.

The 3-year probabilities of death, loss to follow-up and serious morbidity were 7%, 16%, and 28%, respectively (Figure 1). We recorded 47 deaths overall. Of these deceased patients, 15 had signs or symptoms recorded at their last clinic visit. These were nonspecific signs or symptoms leading to: at least one day at the day care hospital (6 patients), severe anemia leading to transfusion (4 patients), hyperlactatemia (2 patients), mediastinal mass on chest radiograph (1 patient), pulmonary TB (1 patient) and enteritis with dehydration (1 patient). The other 32 deceased patients did not show specific symptoms at their last contact with the care team.

## Discussion

We describe serious events that occurred in 1008 adults after ART initiation, in a context of routine care in a country with limited resources. This study was performed in a primary care center with limited resources, thus reflecting what physicians in the field actually diagnose and treat, in a context where patients have to pay for all tests and drugs other than ART monitoring tests and drugs [17].

In this context, we probably underestimated the incidence of severe morbidity, due to the high rate of loss to follow-up, and we might have also misdiagnosed some episodes, due to the lack of paraclinical tests. Unfortunately, these limitations depict an accurate picture of daily clinical practice in most health centres in Côte d’Ivoire.

The most important group of serious morbidity consisted of infectious diseases, malaria, TB, and invasive bacterial diseases. This first group clearly illustrates the burden of common infectious diseases in our population [8,18]. These diseases are both communicable diseases and opportunistic diseases, meaning that they are frequent in the general population, more frequent in HIV-infected patients, and of increasing incidence with decreasing CD4 count [13,19,20].

The second group of serious morbidity consisted of grade 3 or 4 classifying adverse events and non-classifying diseases with hospital stay. Even if all the episodes recorded in this group were not all directly attributable to ART drugs, they illustrate the spectrum of signs and symptoms that clinicians see daily. Physicians constantly have to face the difficult problem of distinguishing serious side-effects—which have direct implication regarding drug discontinuation—from a wide range of episodes including IRIS and non-specific episodes [21-23]. In Côte d’Ivoire, antiretroviral drugs are free of charge, but the costs of transportation, paraclinical investigations, and any non-antiretroviral drugs other than cotrimoxazole are charged to patients [17]. As a result, a significant proportion of serious events remain undiagnosed or untreated because patients cannot afford the care they need. The full consequences of this problem, in terms of avoidable death and avoidable loss-to-follow-up, remain to be investigated.

All recorded events, with the notable exception of malaria, and unfavorable outcomes (death and loss to follow-up) were significantly more frequent in patients with a CD4 count <100/mm^3^ and occurred within the first year following ART initiation, as described previously [7,24,25]. This suggests that starting ART earlier, as recommended by the WHO, would both improve ART outcomes and alleviate the everyday difficulties of dealing with patients who delay treatment and start ART in routine primary care centers in which patients and physicians lack the resources to adequately deal with serious clinical pictures.

## Conclusions

In conclusion, among these HIV-infected adults starting ART and followed in routine conditions, the incidence of serious morbidity was high, especially during the first year on ART, and consisted partly of non-specific episodes with no clearly documented aetiology.

Providing care centers with diagnostic tools and starting ART earlier are necessary steps to improve the quality of HIV care in sub-Saharan primary care facilities.

## Competing interests

The authors declare that they have no competing interests.

Selected data were presented at the 5^eme^ Conférence Francophone VIH/SIDA, Casablanca, Morocco, in March 2010.

## Authors’ contributions

YA, AM, CL, and XA designed the study and wrote the manuscript. YA collected the data and performed the statistical analysis. SE, CD, TO, LD, and AT contributed in the study design and manuscript writing. All authors read and approved the final version of the manuscript.

## Pre-publication history

The pre-publication history for this paper can be accessed here:

http://www.biomedcentral.com/1471-2334/13/607/prepub
